# Endocrine therapy for endometrial cancer: traditional approaches and novel targets

**DOI:** 10.3389/fonc.2025.1570011

**Published:** 2025-05-05

**Authors:** XiaoJing Guan, RongRong Tang, JianHua Yang

**Affiliations:** Department of Gynecology and Obstetrics, Sir Run Run Shaw Hospital, Zhejiang University School of Medicine, Zhejiang, Hangzhou, China

**Keywords:** endometrial cancer, endocrine therapy, traditional targets, novel targets, mechanisms

## Abstract

Recently, the global incidence of endometrial cancer is increasing. Endocrine therapy offers advantages in the management of this malignancy due to its broad applicability and favorable tolerability profile. Although conventional endocrine treatments, including progesterone, gonadotropin-releasing hormone agonists and aromatase inhibitors demonstrate efficacy in endometrial cancer, their long-term utility is limited by adverse effects such as drug resistance and disease recurrence with prolonged treatment. Novel endocrine therapeutic agents, including selective estrogen receptor modulators, selective estrogen receptor degraders, epigenetic-targeted therapies, mTOR inhibitors, cyclin-dependent kinase inhibitors, and metformin, remain in preclinical development or clinical trials. Inspiringly, the preliminary findings suggest these emerging agents may positively impact survival outcomes in endometrial cancer patients. This review examines the mechanisms, methodologies, and efficacy of both traditional and novel endocrine therapeutic approaches for endometrial cancer.

## 1 Introduction

Endometrial cancer (EC) comprises a group of malignancies originating from the endometrial epithelium and represents the most common gynecologic malignancy in developed countries (1). The rising incidence of EC is associated with multiple factors, including population aging, obesity, type 2 diabetes mellitus, female infertility, delayed menopause, and declining rate of hysterectomy for benign conditions (2, 3). Based on molecular analyses from the Cancer Genome Atlas (TCGA) and the Proactive Molecular Risk Classifier for EC (ProMisE), ECs are now classified into four molecular subgroups: POLE-mutated, p53 wild type (low copy number-CNL or nonspecific molecular profile-NSMP), p53 null/missense mutations (high copy number), and mismatch repair deficient (MMRd). This molecular classification has optimized clinical management of EC by defining distinct risk categories (4). Endocrine therapy, representing an early form of targeted therapy for EC, plays a crucial role in fertility-sparing approaches for early-stage disease and non-surgical management of advanced or recurrent EC (5, 6). The NSMP type predominantly manifests as low-grade, early-stage tumors with high expression of estrogen receptor (ER) and progesterone receptor (PR), demonstrating favorable response to endocrine therapy (7). However, the efficacy of monotherapy is usually limited, and resistance often develops with extended treatment cycles. Combination strategies incorporating other targeted therapies or chemotherapy may enhance quality of life and survival outcomes (8).

## 2 Endocrine therapeutic targets for endometrial cancer

### 2.1 Mechanisms of estrogen action

Based on clinical, endocrine and epidemiological characteristics, EC is classified into type I (estrogen-dependent) and type II (non- estrogen-dependent). Type I predominantly comprises endometrioid carcinoma, accounting for 80-90% of all EC cases (9). Intracellular estrogens include estrone (E1), estradiol (E2), and estriol (E3). In premenopausal women, E1 and E2 are primarily secreted by the ovaries, with minor contributions from adipose tissue and the adrenal glands, while E3 is predominantly produced by placental tissue during pregnancy. Ovarian estrogen secretion is regulated by the hypothalamic-pituitary-ovarian (HPO) axis. The hypothalamus secretes gonadotropin-releasing hormone (GnRH), which stimulates the synthesis of luteinizing hormone (LH) and follicle-stimulating hormone (FSH). LH promotes androgen production, which is subsequently aromatized to estrogen by aromatase. Elevated estrogen levels create negative feedback on the hypothalamus and pituitary, inhibiting further estrogen production and secretion (10). Estrogen mediates its biological effects in target tissues by binding to specific receptors, including ERα, ERβ, and G protein-coupled estrogen receptor (GPER) (11). ERα has been implicated in abnormal proliferation, inflammation, and malignant transformation. Estrogen binding to ERα activates PI3K/AKT/mTOR signaling with RAS/RAK/MEK/ERK signaling pathway, directly affecting cyclin D and cyclin-dependent kinase 4/6 (CDK4/6) (12). ERβ may counteract these pro-tumorigenic effects by modulating the expression of ERα-regulated genes (13).

### 2.2 Mechanism of progesterone action

Progesterone, another principal reproductive hormone secreted by the ovaries, plays a critical role in in the differentiation of endometrium. When elevated estrogen levels induce excessive endometrial proliferation without progesterone-mediated differentiation, the endometrium undergoes significant thickening and may develop endometrial atypical hyperplasia (EAH), a precancerous lesion (10, 14). Research indicates that progesterone exerts anti-estrogenic effects through dual mechanisms: it reduces the quantity of estrogen receptors while decreasing the production of new receptors, and it modulates enzymatic activity by reducing the activity of sulfatase and stimulating sulfotransferase, thereby counteracting estrogen production (15).

### 2.3 Endocrine therapeutic targets for endometrial cancer

**Figure 1:**
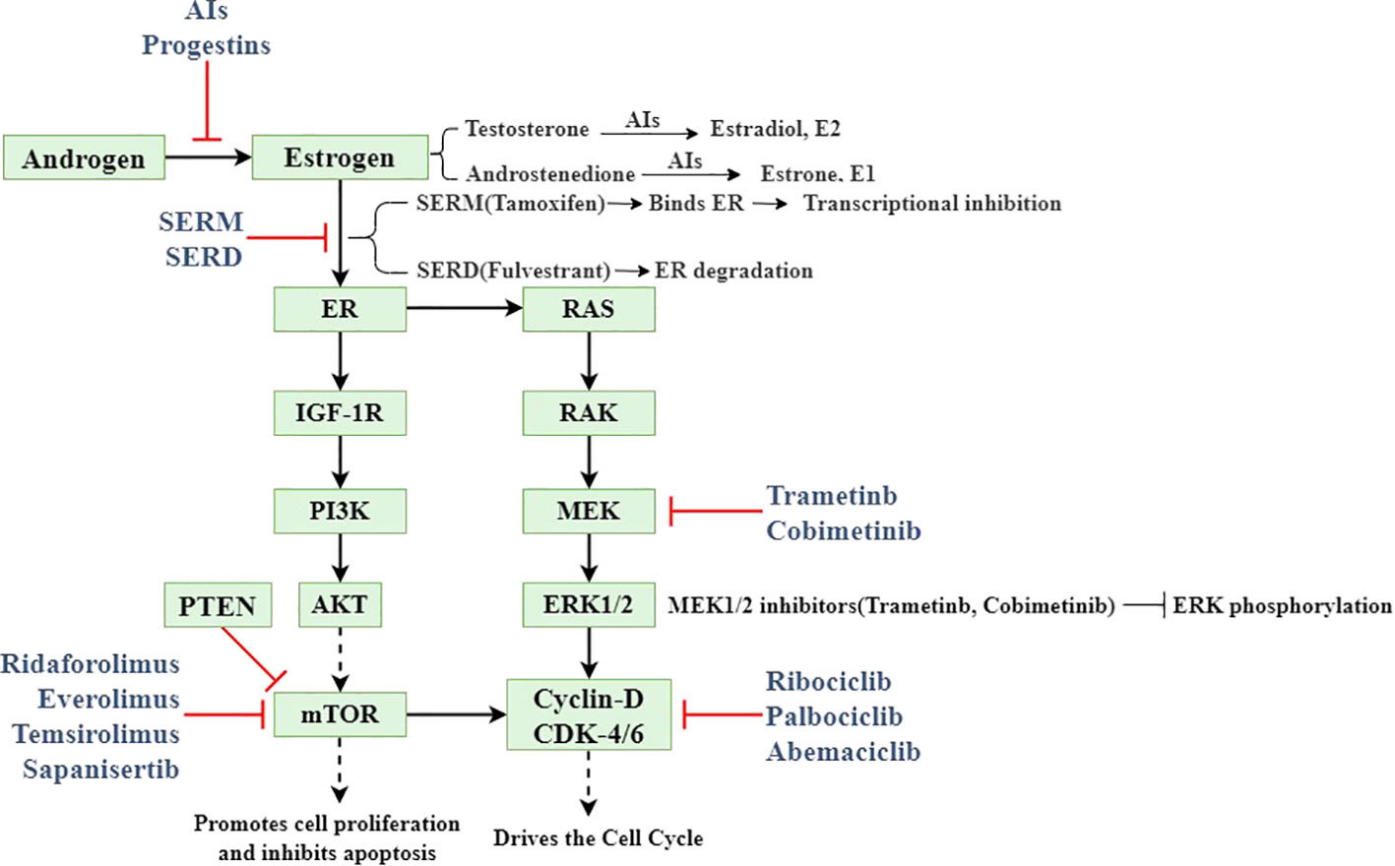
Model diagram of the mechanism of endocrine therapy in endometrial cancer.

Multiple therapeutic approaches target the estrogen production pathway. Gonadotropin-releasing hormone agonists (GnRHα) and aromatase inhibitors (AIs) effectively reduce estrogen production, thereby mitigating EC progression (16). Oral progestins and/or levonorgestrel intrauterine systems (LNG-IUS) represent the primary modalities for progesterone supplementation in EC management, particularly for EAH and early-stage EC, offering effective fertility preservation (17). To prevent estrogen-receptor binding, selective estrogen receptor modulators (SERMs) regulate ER activity by competing with estrogen for ER binding and altering associated cofactors. Additionally, selective estrogen receptor degraders (SERDs) destabilize the estrogen receptor H12 domain upon binding, inducing ER degradation (18). Epigenetic approaches, including histone deacetylase (HDAC) inhibitors, DNA methylation (DNMT) inhibitors, histone methyltransferases (HMTs), and histone demethylases (HDMs), effectively alter the transcription of key genes affecting EC cell proliferation, apoptosis, and receptor expression of estrogen and progesterone (19). EC exhibits the highest frequency of mutations in the PI3K/AKT/mTOR pathway compared to other tumor types in TCGA, suggesting that inhibitors targeting this pathway may benefit EC patients (20, 21). Preclinical studies demonstrate that cyclin-dependent kinase inhibitors (CDKis) may address endocrine therapy resistance partially mediated by PI3K overactivation; however, additional clinical data regarding the efficacy and adverse effects of CDKis in EC patients are needed (22). The above mechanism of action and targets are shown in **Figure 1**. Additional endocrine therapeutic agents include metformin, androgen receptor (AR) antagonists, and steroid sulfatase (STS) etc. The key endocrine therapeutic agents with their respective mechanisms and molecular targets are systematically summarized in **Table 1**.

**Table 1:** Summary of endocrine therapeutic agents in endometrial cancer: mechanisms and targets.

Therapeutic Category	Representative Agents	Mechanism of Action	Molecular Targets
Traditional Agents			
Progestins	MA, MPA, LNG-IUS	Bind to progesterone receptors to induce endometrial differentiation	Progesterone receptor (PR)
GnRH agonists	Leuprolide, Goserelin, Triptorelin	Downregulate pituitary gonadotropin secretion, suppress ovarian estrogen production	GnRH receptor
Aromatase inhibitors	Anastrozole, Letrozole	Inhibit aromatase-mediated conversion of androgens to estrogens	Aromatase
Novel Agents			
SERMs	Tamoxifen	Compete with estrogen for ER binding, modulate ER transcriptional activity	ERα/ERβ
SERDs	Fulvestrant	Induce ER conformational changes leading to receptor degradation	ERα/ERβ
HDAC inhibitors	Entinostat	Inhibit histone deacetylation, restore tumor suppressor gene expression	HDAC1/2/3
mTOR inhibitors	Everolimus, Sapanisertib, Ridaforolimus, Temsirolimus	Block PI3K/AKT/mTOR signaling pathway	mTOR
CDKis	Ribociclib, Abemaciclib	Inhibit cyclin-dependent kinases, induce cell cycle arrest	CDK4/6
Metformin	Metformin	Activate AMPK pathway, inhibit PI3K/AKT/mTOR signaling	AMPK, PI3K/AKT/mTOR
Combination Therapies			
AI + mTOR inhibitor	Letrozole + Everolimus	Dual suppression of estrogen synthesis and PI3K pathway	Aromatase & mTOR
Progestin + HDAC inhibitor	MPA + Entinostat	Synergistic activation of PR signaling and epigenetic modulation	PR & HDAC
GnRHα + LNG-IUS	Goserelin + Levonorgestrel	Systemic estrogen suppression combined with local progesterone delivery	GnRH receptor & PR

MA, medroxyprogesterone; MPA, medroxyprogesterone acetate; LNG-IUS, levonorgestrel intrauterine systems; PR, progesterone receptor; GnRHα, gonadotropin-releasing hormone agonists; AIs, aromatase inhibitors; SERM, selective estrogen receptor modulator; ER, estrogen receptor; SERD, selective estrogen receptor degrader; HDAC, histone deacetylase; mTOR, mammalian target of rapamycin; PI3K, phosphatidylinositol 3-kinase; AKT, protein kinase B; CDKis, cyclin-dependent kinase inhibitors; AMPK, adenosine monophosphate activated protein kinase.

## 3 Endocrine therapy for endometrial cancer

### 3.1 Progesterone

Progestogens were the initial agents clinically employed in endocrine management of EC. Oral progestins, primarily medroxyprogesterone (MA) and medroxyprogesterone acetate (MPA), represent the standard approach. In early-stage EC, oral long-acting MPA reduces tumor proliferation, promotes tumor differentiation, and provides benefit during the preoperative interval (23). LNG-IUS, an intrauterine sustained-release progestin system, has gained acceptance among gynecologists due to its localized action, minimal systemic adverse effects, and high patient compliance. Gallos et al. (24) demonstrated that LNG-IUS may yield superior clinical outcomes compared to oral progestins. Janda et al. (25) reported a 61% pathological complete response rate following 6 months of LNG-IUS treatment in early-stage EC. Similarly, Westin et al. (26) observed a 12-month clinical remission rate of 83% with LNG-IUS in the treatment of EAH and early-stage EC, including 90.6% for EAH and 66.7% for early-stage EC. Despite the satisfactory short-term outcomes of progesterone therapy in EAH and early-stage EC, preventing drug resistance and disease recurrence remains a critical challenge. Research has documented recurrence rates as high as 30-40% following progesterone therapy for early-stage EC, potentially associated with PR-A and PR-B expression ratios in endometrial glands and stroma. Women with PR-A:PR-B ≤ 1 exhibited a higher recurrence risk than those with PR-A:PR-B > 1 (26, 27). Multiple clinical trials suggest beneficial effects of progestins in combination with GnRHα, HDAC inhibitors, and metformin for the treatment of EAH and early-stage EC and for recurrence prevention, detailed in subsequent sections. In conclusion, oral progestins and/or LNG-IUS effectively alleviate clinical symptoms and pathological features of EAH and early-stage EC. However, therapeutic effects are often short-lived, with prolonged use associated with drug resistance and disease recurrence. Future therapeutic strategies will focus on overcoming drug resistance through combination approaches to prevent EC progression and recurrence.

### 3.2 GnRHα

GnRH is a decapeptide secreted by the hypothalamus that regulates pituitary gonadotropin secretion. In preclinical models, GnRH-I and GnRH-II agonists, antagonists, and cytotoxic GnRH-I analogs have demonstrated antiproliferative effects and apoptosis induction in human EC cell lines (28). Several studies from the 1990s established that GnRHα exhibits significant antitumor activity against recurrent EC while effectively alleviating pain in EC patients (29, 30). However, subsequent in-depth investigations revealed that single-agent GnRHα therapy (leuprolide, goserelin, and triptorelin) demonstrated low response rate and limited efficacy in recurrent EC management (31–33). In the study by Asbury et al., a positive therapeutic response was observed in a patient who had previously received hormone therapy, despite the limited efficacy of single-agent goserelin acetate in recurrent EC (32). This finding suggests that GnRHα may enhance therapeutic efficacy in EC through combination with other endocrine therapies. GnRHα combined with LNG-IUS or AIs demonstrates efficacy in treating EAH and early-stage EC and represents a viable alternative to hysterectomy for fertility preservation (34, 35).

### 3.3 AIs

AIs interfere with endogenous estrogen production by inhibiting aromatase activity and may be employed in EC management. In a randomized trial evaluating anastrozole as a neoadjuvant therapeutic agent, significant reductions in ER, AR, and Ki67 expression in endometrial glands were observed following 14 days of anastrozole treatment, suggesting potential benefits of preoperative anastrozole in EC management (36). For elderly patients ineligible for standard surgical intervention, early administration of oral anastrozole may alleviate or stabilize disease and improve quality of life (37). Mileshkin et al. (38) conducted a phase II clinical trial assessing the efficacy of anastrozole in ER/PR-positive recurrent/metastatic EC. Although the objective response rate to monotherapy was relatively low, nearly half of the patients derived clinical benefit from treatment, and the degree of anastrozole benefit did not strongly correlate with ER/PR expression levels. Conversely, Lindemann et al. (39) found that ER-positive patients treated with the AIs exemestane demonstrated significantly higher rates of disease remission, median progression-free survival (PFS), and overall survival (OS) compared to ER-negative patients in a phase II clinical trial of ER-positive advanced/recurrent EC. To enhance efficacy, AIs are frequently combined with other agents in clinical practice. Beyond the previously mentioned combination with GnRHα for fertility-preserving treatment of EAH and early-stage EC, AIs combined with mTOR inhibitors have shown promise in advanced/recurrent EC management and survival prolongation. In summary, AIs monotherapy demonstrates greater efficacy in EAH and early-stage EC, with more limited efficacy in advanced or recurrent disease. The relationship between therapeutic efficacy and ER/PR status requires further investigation (40). Additionally, AIs combination with other agents (e.g., GnRHα and mTOR inhibitors) may enhance EC therapeutic efficacy and compensate limitations of monotherapy.

### 3.4 SERM and SERD

Tamoxifen, the most commonly utilized SERM analogue in clinical practice, exhibits dual effects in EC development and treatment. On one hand, tamoxifen use for other conditions increases EC risk by 1.5-6.9 fold; on the other hand, tamoxifen demonstrates utility in advanced/recurrent EC management (41). Tamoxifen may exert these effects by functioning as an agonist or antagonist through ERα, depending on cellular variations in coactivators or corepressors (42). Advanced/recurrent EC management frequently involves tamoxifen in combination with progesterone to counteract PR downregulation and enhance the antitumor activity of progesterone (8). Fulvestrant, a SERD analogue, possesses estrogen receptor antagonistic property, but its efficacy in EC remains uncertain. Results from two available phase II clinical trials suggest limited activity of fulvestrant in recurrent/metastatic EC management. Additionally, fulvestrant demonstrates poor bioavailability, and optimal therapeutic dosing remains undetermined (43–45). Combination approaches with fulvestrant may enhance therapeutic efficacy in EC; however, new clinical trials have not been conducted, and fulvestrant has not received approval for EC treatment.

### 3.5 Targeted epigenetics

Since Hrzenjak et al. (46) initially reported significantly higher HDAC2 expression in endometrial stromal sarcomas (ESS) compared to normal endometrial stroma, subsequent studies have demonstrated significantly elevated expression of HDAC1, HDAC2, and HDAC3 in EC (47). *In vitro* studies have shown that HDAC inhibitors effectively induce cell cycle arrest, reduce cell proliferation, and increase p21 expression in EC. Additionally, HDAC inhibitors enhance PR expression, which regulates cellular differentiation and exerts important anticancer effects (48). Duska et al. (49) conducted a clinical trial evaluating the HDAC inhibitor entinostat in combination with MPA during the surgical window. Results indicated that entinostat did not directly affect the expression of endometrial PR in the short term; however, Ki67 expression levels were lower with combination therapy compared to MPA monotherapy. In summary, HDAC inhibitors demonstrate promise in EC management. However, additional trials are necessary to determine the therapeutic role, utility, and safety profile of these agents. In addition, several studies have demonstrated correlations between DNA and histone methylation with EC development, although investigations have been limited to *in vitro* experiments (19).

### 3.6 mTOR inhibitors

mTOR inhibitors target diseases with PI3K/AKT/mTOR pathway mutations and are currently undergoing clinical trials for EC therapy. Based on several clinical investigations, mTOR inhibitors such as everolimus, sapanisertib, ridaforolimus, and temsirolimus have demonstrated preliminary antitumor activity in advanced/recurrent EC management (50–53). Beyond monotherapy, mTOR inhibitors combined with AIs show particular promise in EC treatment. Heudel et al. (54) found that adding the mTOR inhibitor vistusertib to anastrozole further prolonged 8-week PFS and median PFS and improved overall response rates in EC patients. The combination of everolimus and letrozole demonstrates high clinical benefit rates (CBR) and objective response rates (ORR) in patients with recurrent/progressive EC, with particularly notable efficacy in endometrioid EC harboring *CTNNB1* mutations (55).

### 3.7 CDKis

Both the inherent endocrine characteristics of EC and its manifestation as a cell cycle dysregulation suggest potential utility for CDKis in these patients. Colon-Otero et al. (56) hypothesized that CDKis combined with AIs might be suitable for EC management based on the efficacy of this approach in breast cancer. Using clinical samples and patient-derived xenograft (PDX) models, they demonstrated that ribociclib (a CDKi) combined with letrozole produced positive survival effects in patients with recurrent ER-positive EC. Similarly, Konstantinopoulos et al. (57) found that letrozole combined with abemaciclib (a CDKi) demonstrated positive response effects in recurrent/metastatic EC, independent of tumor grade, prior hormone therapy, mismatch repair status, and PR status.

### 3.8 Metformin

Metformin, initially developed for diabetes management, has increasingly demonstrated importance in oncology. Multiple clinical trials have shown that metformin administration during the EC surgical window reduces Ki67 expression in tumor tissue while altering the phosphorylation status of PI3K/AKT/mTOR pathway proteins (58–60). However, Kitson et al. (61) demonstrated in a phase III clinical trial that short-term metformin treatment did not reduce Ki67 expression in EC tissues and failed to demonstrate positive effects on tumor proliferation inhibition. In combination approaches, metformin appears to offer superior efficacy compared to MA monotherapy in EAH management when used in combination with MA (62, 63). Additionally, in EAH and early-stage EC, metformin may inhibit disease recurrence following MPA treatment and contribute to fertility preservation (64).

## 4 Conclusion

Traditional endocrine therapy maintains a significant role in EC fertility preservation, preoperative neoadjuvant therapy, and palliative care for advanced disease; however, challenges such as high rates of treatment resistance and recurrence require continued attention. Novel therapeutic approaches may address limitations of conventional treatments, but research on emerging agents remains relatively limited, most still in preclinical development, resulting in insufficient clinical data regarding efficacy and adverse effects. Future research will continue to focus on novel targets and therapeutic modalities. Concurrently, combinations of traditional and novel agents may yield enhanced outcomes for EC patients.
